# The electronic stethoscope

**DOI:** 10.1186/s12938-015-0056-y

**Published:** 2015-07-10

**Authors:** Shuang Leng, Ru San Tan, Kevin Tshun Chuan Chai, Chao Wang, Dhanjoo Ghista, Liang Zhong

**Affiliations:** National Heart Research Institute Singapore, National Heart Centre Singapore, 5 Hospital Drive, Singapore, 169609 Singapore; Cardiovascular and Metabolic Disorders Program, Duke-NUS Graduate Medical School, 8 College Road, Singapore, 169857 Singapore; Institute of Microelectronics, A*STAR, 11 Science Park Road, Singapore, 117685 Singapore; University 2020 Foundation, Northborough, MA USA

**Keywords:** Heart sound, Heart auscultation, Heart disorder, Diagnosis, Acoustic technique, Automatic system, Smartphone stethoscope apps

## Abstract

Most heart diseases are associated with and reflected by the sounds that the heart produces. Heart auscultation, defined as listening to the heart sound, has been a very important method for the early diagnosis of cardiac dysfunction. Traditional auscultation requires substantial clinical experience and good listening skills. The emergence of the electronic stethoscope has paved the way for a new field of computer-aided auscultation. This article provides an in-depth study of (1) the electronic stethoscope technology, and (2) the methodology for diagnosis of cardiac disorders based on computer-aided auscultation. The paper is based on a comprehensive review of (1) literature articles, (2) market (state-of-the-art) products, and (3) smartphone stethoscope apps. It covers in depth every key component of the computer-aided system with electronic stethoscope, from sensor design, front-end circuitry, denoising algorithm, heart sound segmentation, to the final machine learning techniques. Our intent is to provide an informative and illustrative presentation of the electronic stethoscope, which is valuable and beneficial to academics, researchers and engineers in the technical field, as well as to medical professionals to facilitate its use clinically. The paper provides the technological and medical basis for the development and commercialization of a real-time integrated heart sound detection, acquisition and quantification system.

## Background

Heart disease is the leading cause of death in most countries in the world. In 2012, cardiovascular diseases killed 17.5 million people, i.e., three in every ten deaths [1]. The valvular heart disease (VHD) is one typical type of cardiovascular disease causing significant indisposition and adverse effect on the functionality and long term life of the patient [2]. In the reduction of deaths from heart diseases (particularly VHD), diagnosis plays a vital role. The electrocardiogram (ECG) is a powerful and common screening tool for heart diseases. It is relatively inexpensive, non-invasive, and easy to use. However, it does have some limitations, one of which is the difficulty in detecting structural abnormalities in heart valves and defects characterized by heart murmurs [3]. Nowadays, the magnetic and ultrasound scanner can take detailed and even moving images of the heart. Echocardiogram (echo) uses the principle of rebounding waves to create a moving picture of the heart. It provides information about the size, shape, structure and function of the heart. Cardiac magnetic resonance imaging (MRI) uses radio waves, magnets and a computer to create pictures of the heart as it beats. The MRI test produces both still and moving pictures of the heart and major blood vessels [4]. A computed tomography (CT) scan of the heart, on the other hand, is an imaging method that uses x-rays to create detailed pictures of the heart and its blood vessels. Recently, many advanced geometry processing techniques have been developed for the MRI and CT based 3D and 4D heart model reconstruction, to further understand and visualize what we are unable to obtain from static 2D images [5–8]. However, the major disadvantages of echo, MRI and CT are their high cost and the need of specialized personnel to operate the complex machines. These equipments are usually only affordable in large hospitals in the big cities. According to WHO [9], nearly 80% of deaths due to cardiovascular disease occur in low- and middle-income countries. The availability of the medical imaging equipments in these countries is quite low.

Therefore, it is very important to have a cost effective and accurate method for the early detection of cardiac illnesses. Heart auscultation, defined as listening and interpretation of the heart sound (HS), has been a very important method for the early diagnosis of heart diseases [10] by capturing abnormal HSs. A phonocardiogram (PCG) is a plot of high fidelity recording of the HS with the help of the machine called phonocardiograph. The HS and PCG are often used interchangeably in the literature. Throughout our study, unless otherwise stated, the term HS is used. Despite the advantages of low cost and easy operation, auscultation of the heart has been traditionally limited by three factors. Firstly, as the HS contains a mixture of high frequency (HF) and low frequency (LF) acoustic signals with low amplitude, it is highly required for the stethoscope (or the sensor used in the stethoscope) to have a high selective sensitivity. Secondly, the HS data recorded with the stethoscope is often corrupted with noise, which can prohibit accurate and effective diagnosis of heart diseases. Thirdly, the interpretation of the HS is very subjective, and it depends largely on the experience, skills, and hearing ability of the physician. Therefore, the need for advancement of heart auscultation is highly evident.

The emergence of electronic stethoscope has opened a new field named “computer-aided auscultation”. With the recent developments in the technology, from acoustic sensor design, advanced digital signal processing to the computer based machine learning techniques, the acoustic based automatic diagnosis of cardiac dysfunction by electronic stethoscope has attracted much attention in recent years. This paper provides a comprehensive review of literature articles, market (state-of-the-art) products, and smartphone stethoscope apps covering every key component of computer-aided cardiac dysfunction detection system using the electronic stethoscope. We believe that the information provided in this paper will be valuable and beneficial to not only the researchers and engineers in the technical field, but also to medical professionals.

This paper is organized as follows. The characteristics of the HS are first discussed. The paper subsequently gives the overall system design and the organization of the review. The detailed results of the review study are then presented, followed by discussions and recommendations. Finally concluding remarks are reported.

## Characteristics of heart sounds

**Figure 1:**
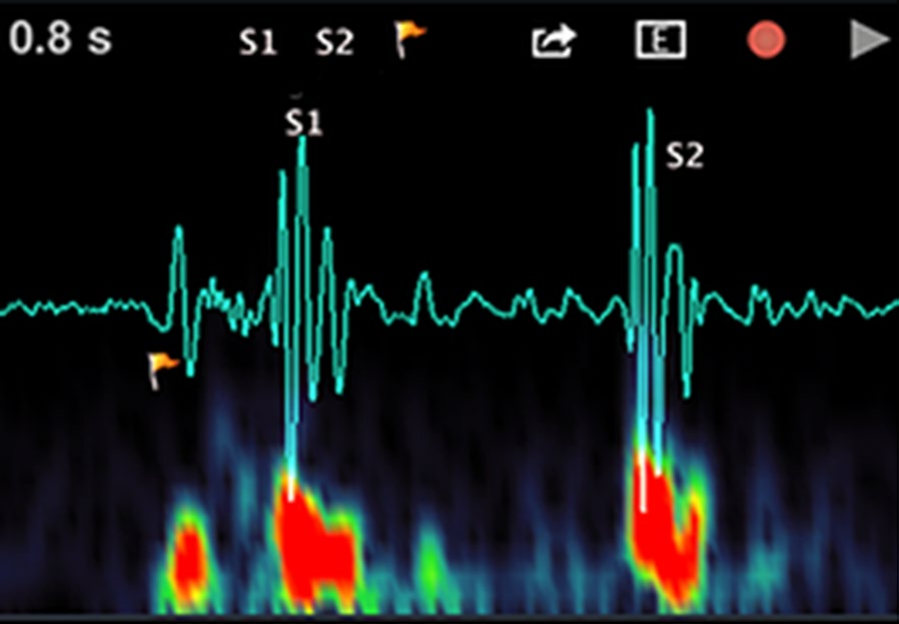
Two primary heart sounds: S1 and S2.

HSs are generated by the beating heart and the resultant flow of blood through it [11]. In healthy adults, there are two normal HSs (as illustrated in Figure 1): the first HS (S1), produced by the closing of the atrioventricular valves; and the second HS (S2), caused by the closure of the semilunar valves. In the case of abnormal HS, there could be other several signal activities between S1 and S2 such as S3, S4, murmur, etc. The third HS (S3) is a rare extra sound caused by a sudden deceleration of blood flow into the left ventricle from the left atrium. This sound is normal in children and adults up to age 35–40 years. After the age of 40, a third HS is usually abnormal and correlates with dysfunction or volume overload of the ventricles [12]. The fourth HS (S4) is caused by the vibration of valves, supporting structures and the ventricular walls. S4 is proved to be a sign or symptom of heart failure during diastolic period. In general, the frequency of S1 is lower than that of S2, and the duration of S1 is longer than that of S2. The S3 occurs from 0.1 to 0.2 s after S2, while S4 occurs from 0.07 to 0.1 s before S1—both of them are low pitched. In addition to these HSs, numerous heart murmurs may arise mainly from heart problems or diseases. The murmurs are extra or unusual sound heard during a heartbeat and broadly classified as systolic, diastolic and continuous [13, 14].

**Figure 2:**
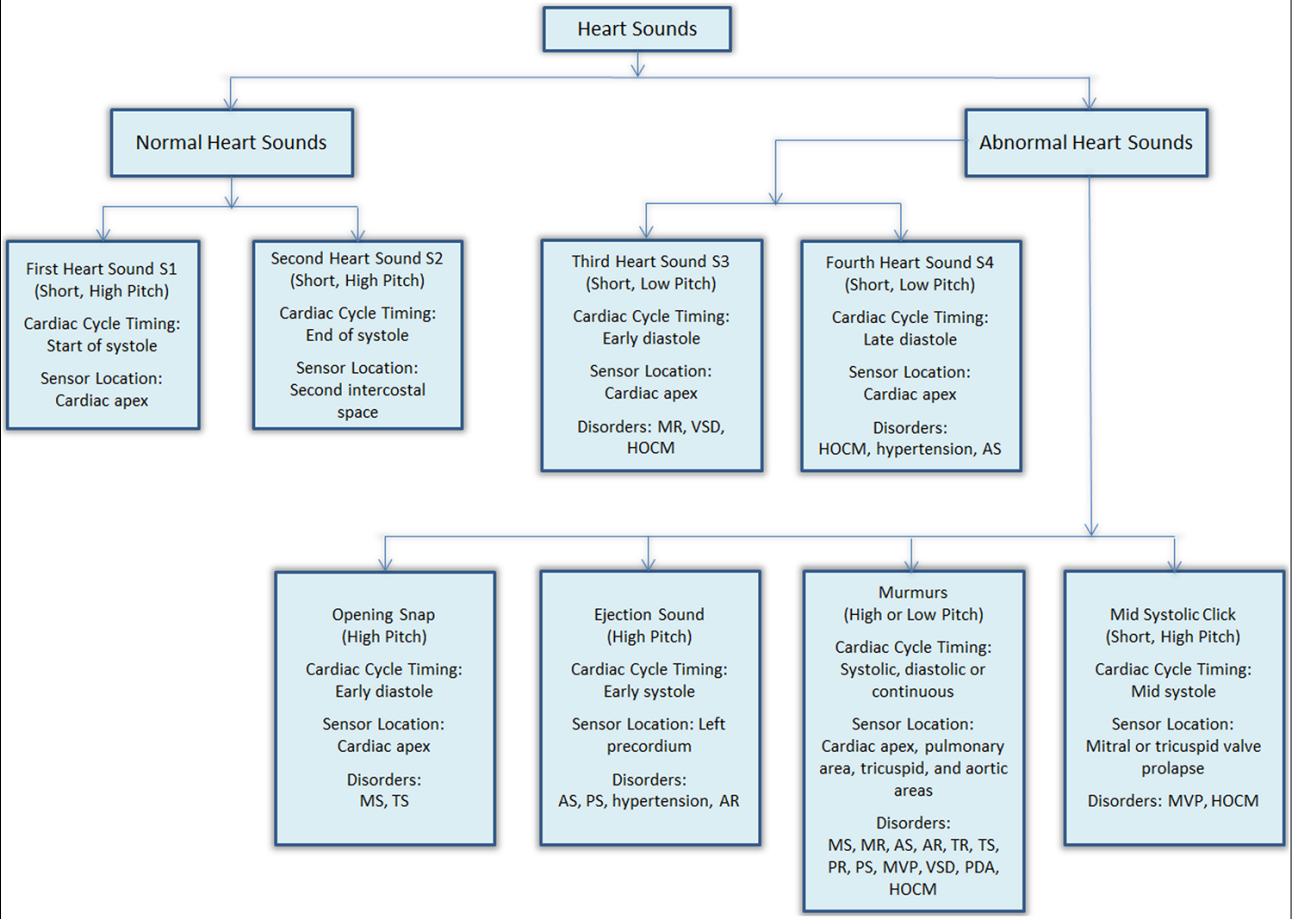
Characteristics of heart sounds.

The complete types and characteristics of the HSs are listed in Figure 2. Each heart disorder is associated with one or more HSs. The disorders that are associated with each sound are also detailed in Figure 2. The summary of characteristics of some common murmurs is provided in Table 1.

**Table 1:** Characteristics of some common murmurs

Murmur type	Location	Timing	Pitch	Quality
Mitral stenosis (MS)	Apex	Diastolic	Low	Rumbling
Mitral regurgitation (MR)	Apex	Systolic	High	Blowing
Aortic stenosis (AS)	Apex/right upper sternal border	Systolic	High	Harsh
Aortic regurgitation (AR)	Right upper sternal border/left third/fourth intercostal space (ICS)	Diastolic	High	Blowing
Tricuspid stenosis (TS)	Lower right and left sternal borders	Diastolic	High	Rumbling
Tricuspid regurgitation (TR)	Left fourth ICS	Diastolic	High	Blowing
Pulmonary stenosis (PS)	Left second ICS	Systolic	High	Blowing
Pulmonary regurgitation (PR)	Second/third ICS	Diastolic	High	Blowing
Mitral valve prolapse (MVP)	Apex	Mid-late systolic	High	Blowing
Ventricular septal defect (VSD)	Left lower sternal border	Systolic	High	Harsh
Patent ductus arteriosus (PDA)	Left upper sternal border	Continuous	High	Harsh
Hypertrophic cardiomyopathy (HOCM)	Left lower sternal border	Mid-late systolic	High	Harsh

In some miscellaneous HSs, the opening snap is a high-pitched diastolic sound produced by rapid opening of mitral valve in mitral stenosis (MS) or tricuspid valve in tricuspid stenosis (TS). The ejection sound (ES) is the most common early systolic sound which results from abnormal sudden halting of the semilunar cusps as they open during early systole. The mid-systolic click (MSC) is a HF sound in mid systole that results from the abrupt halting of prolapsing mitral valve leaflets’ excursion into the atrium by chordae [15].

During cardiac auscultation, the physicians are particularly interested in abnormal sounds, and various types of murmurs, which may suggest the presence of a cardiac pathology and also provide diagnostic information.

## System overview

**Figure 3:**
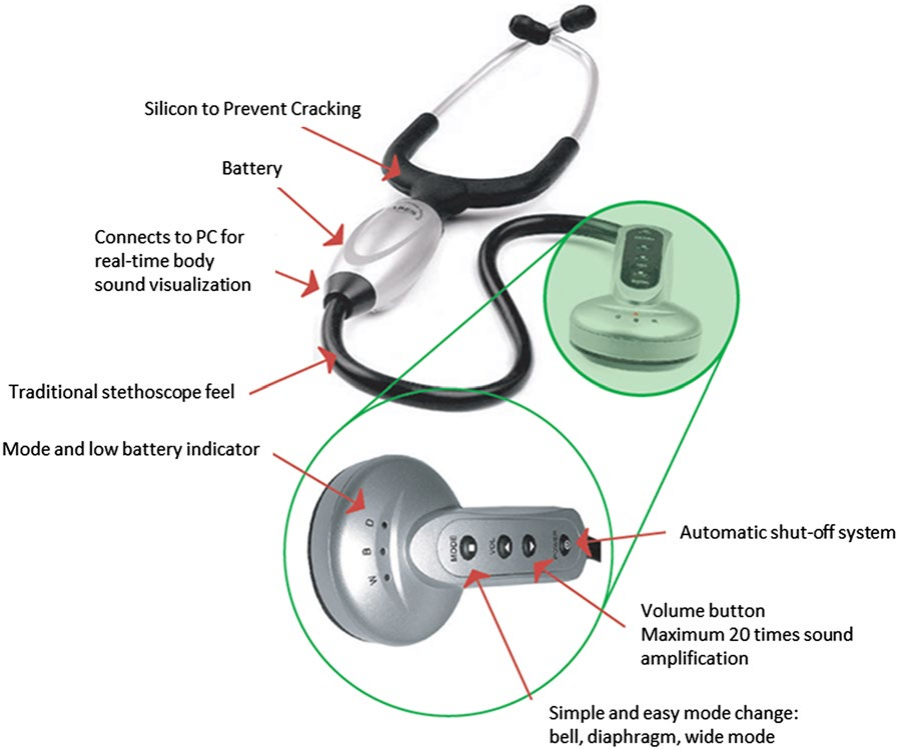
One state-of-the-art electronic stethoscope [16].

Figure 3 shows one example of the state-of-the-art electronic stethoscopes, which provides the choice of bell, diaphragm and wide mode to pick the right frequency for better body sound acquisition. It allows the physician to record the HS of their patients directly onto their PC or laptop for further visualization and analysis. With this capability, healthcare givers can have better understanding and interpretation of the HS for better-quality medical services. More detailed discussion on the commercially available electronic stethoscopes can be found in the later section.

**Figure 4:**
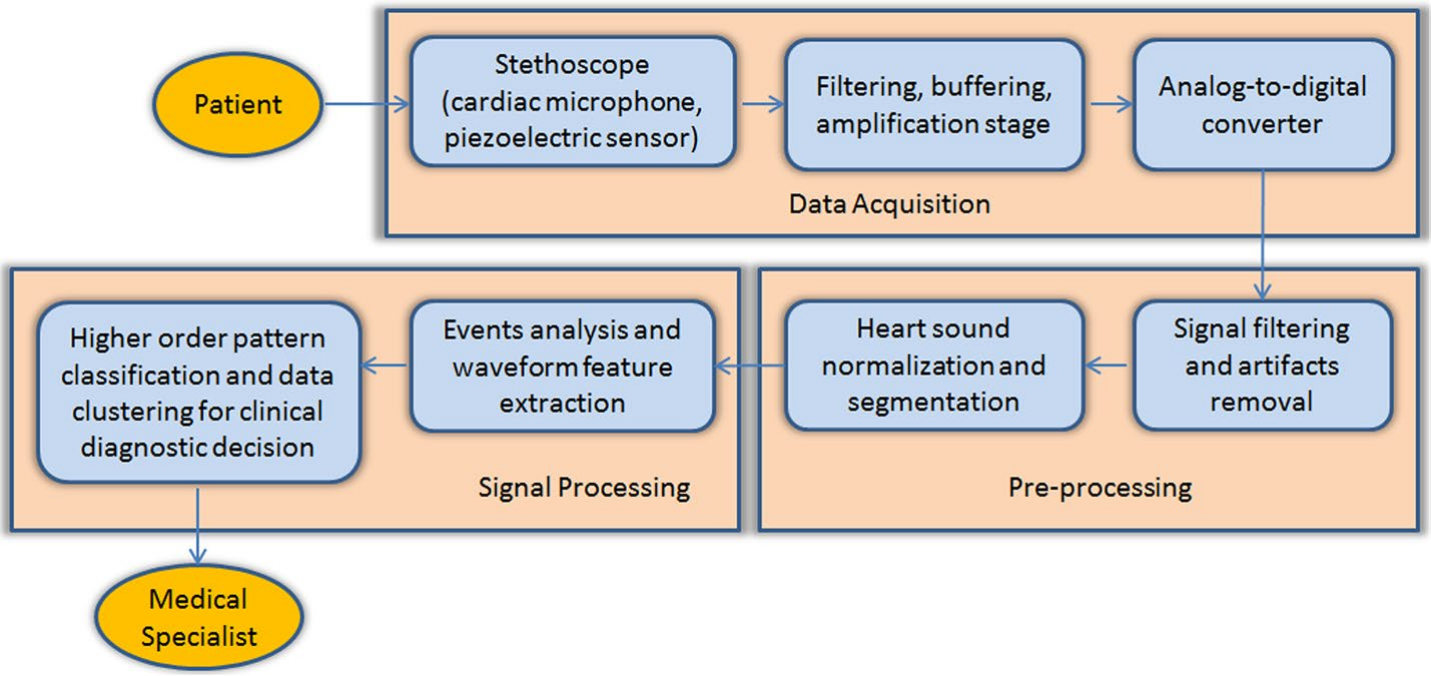
Typical flow chart for heart sound signal acquisition, processing and analysis.

Usually, there are three main modules, namely data acquisition module, pre-processing module and signal processing module, in the computer-based cardiac dysfunction detection system using electronic stethoscope, as displayed in the flow chart of Figure 4 [17]. For the data acquisition module, an electronic stethoscope records the HS and the associated electronics converts it into digital signals and sends it to the pre-processing module. In the pre-processing module, the filtered and interference reduced HS signal is normalized and segmented. Feature extraction and classification are carried out in the signal processing module. The output of the system is the classification result for clinical diagnostic decision making. Detailed descriptions on the three main modules and their sub-modules are provided as follows.

### Heart sound data acquisition module

The HS acquisition stage creates the digital HS data for further processing.

*Electronic stethoscope sensor* The HS signals are directly collected from patients by using an electronic stethoscope. Some commonly used transducers in the stethoscope are microphone, piezoelectric sensors, etc. The sound signals from the heart are converted to analog electrical signals.

*Amplifier and filter* Amplification and filtering are the two major aspects in any signal acquisition system. Usually, a pre-amplifier with a small gain is used to suppress the 50 or 60 Hz interference from power lines. An anti-aliasing filter is then employed to prevent aliasing effect. In some system designs, the filter section is built with a band pass filter circuit having the frequency range of most HS signals. The use of band pass filter with proper passband selection not only prevents aliasing, but also removes some of the noises outside the passband. In post amplification, the filtered signal is amplified to the level range required by the analog-to-digital converter.

*Analog-to-digital converter* The amplified and filtered analog signal is converted to digital signal by the analog-to-digital converter. The sampling frequency and bit resolution can be set by the system designer. Usually, a higher sampling rate and bit resolution will provide greater accuracy, at the cost of more bandwidth required and power consumption.

### Heart sound pre-processing module

In this stage, the digital HS signal will undergo noise reduction, normalization and segmentation.

*Signal denoising unit* A digital filter is sometimes used to extract the signal within the frequency band of interest from the noisy data. In order to equip the system with even better denoising capability, some advanced artifacts removal techniques are generally utilized such that the output signal-to-noise ratio (SNR) can be further improved.

*Normalization and segmentation* In data acquisition, different sampling and acquisition locations normally result in a signal variation. Thus, the HS signals are normalized to a certain scale, so that the expected amplitude of the signal is not affected from the data acquisition locations and different samples. After getting the normalized signals, the HS signals are segmented into cycles which are ready for HS components detection and features extraction.

### Heart sound signal processing module

The feature extraction and classification are conducted in this stage.

*Feature extraction* Signal processing is carried out to convert the raw data to some type of parametric representation. This parametric representation, called feature, is then used for further analysis and processing.

*Classification* A classifier, trained with the extracted features, is used to categorize the data and assist the medical specialist for clinical diagnostic decision making.

Therefore, the processing blocks (shown in Figure 4) form the core units of a computer-aided HS measurement and analysis system. Based on extensive study, we have found that the study for the automated detection of various heart pathological conditions and diseases from HS signal mainly focuses on three stages: (1) HS acquisition system and sensor design (2) denoising and segmentation of HS signals, and (3) appropriate feature extraction and automatic interpretation of HS.

## Organization of review

The review process covers three main aspects, namely articles, state-of-the-art products and smartphone stethoscope apps review.

The purpose of the ‘article review’ is to provide in-depth summary and evaluation of the recent progress in computer-based HS analysis from the engineering perspective. For this purpose, relevant articles were initially identified from searches of various electronic resources, such as IEEE, Springer, Elsevier, PubMed and ACM digital library databases. The search was carried out by using the keyword “heart sound denoising” and “heart sound classification”. A selection criterion was finalized, and every article was selected according to the selection criteria. A total of 21 and 53 articles, respectively, were included in the final selection of articles for HS denoising and classification. Furthermore, some brief studies on the HS recording sensor and HS segmentation methods have also been done, and their summaries are provided for the completeness of the review covering all major components of the overall HS analyzing system.

The objective of ‘market and stethoscope apps review’ is to validate the needs, problems and gaps from the medical and market point of view as well as from a technology push perspective. The prior art search for market and smartphone stethoscope apps was performed by using the web search engines with the keyword “electronic stethoscope” and “stethoscope apps”, respectively. A total of eight products and five apps were selected for analysis and comparison.

The results based on a comprehensive review of (1) Literature Articles, (2) Market (state-of-the-art) Products and (3) Smartphone Stethoscope Apps reviews are presented in the subsequent sections namely “Literature articles review results, Market (state-of-the-art) products review results, Smartphone stethoscope apps review results”.

## Literature articles review results

The literature articles review results consist of four subsections:

- *Sensors for HS recording* in data acquisition module
- *Heart sound denoising* in pre-processing module
- *Heart sound segmentation* in pre-processing module
- *Feature extraction and classificationof HS* in signal processing module

### Sensors for heart sound recording

There are generally two types of stethoscopes: the traditional acoustic stethoscope and electronic stethoscope. The acoustic stethoscope operates on the transmission of sound from the chest piece, via air-filled hollow tubes, to the listener’s ears. The chest piece usually consists of two parts that can be placed against the patient for sensing sound: the bell transmitting LF sounds and the diaphragm transmitting HF sounds. One problem with acoustic stethoscope is that the sound level is extremely low. An electronic stethoscope overcomes the low sound levels by electronically amplifying the body sounds. Electronic stethoscopes convert the acoustic sound waves obtained through the chest piece into electrical signals which can then be amplified for optimal listening. The converted electrical signals can also be digitalized for further processing and transmission. It has been shown that compared with the conventional stethoscope, the electronic stethoscope is considered to be better for hearting HSs, even though no benefit can be found for breathing sounds [18].

Unlike acoustic stethoscopes, transducers or sensors in electronic stethoscopes vary widely. The simplest method of sound detection is achieved by placing a microphone in the chest piece. The microphone, mounted behind the stethoscope diaphragm, picks up the sound pressure created by the stethoscope diaphragm, and converts it to electrical signals. The microphone itself has a diaphragm, and thus the acoustic transmission path comprises of the stethoscope diaphragm, air inside the stethoscope housing, and finally the microphone diaphragm. The existence of two diaphragms and the intervening air path result in excess ambient noise pickup by the microphone, as well as inefficient acoustic energy transfer [19].

The piezo-electric sensors operate on a somewhat different principle than merely sensing diaphragm sound pressure [20–24]. Piezo-electric sensors produce electrical energy by deformation of a crystal substance. In one case, the diaphragm motion deforms a piezoelectric sensor crystal, which is mechanically coupled to the stethoscope diaphragm, and an electrical signal results. The problem with this sensor is that the conversion mechanism produces signal distortion compared with sensing the pure motion of the diaphragm. The resulting sound is thus somewhat different in tone and distorted compared with that obtained by an acoustic stethoscope.

**Figure 5:**
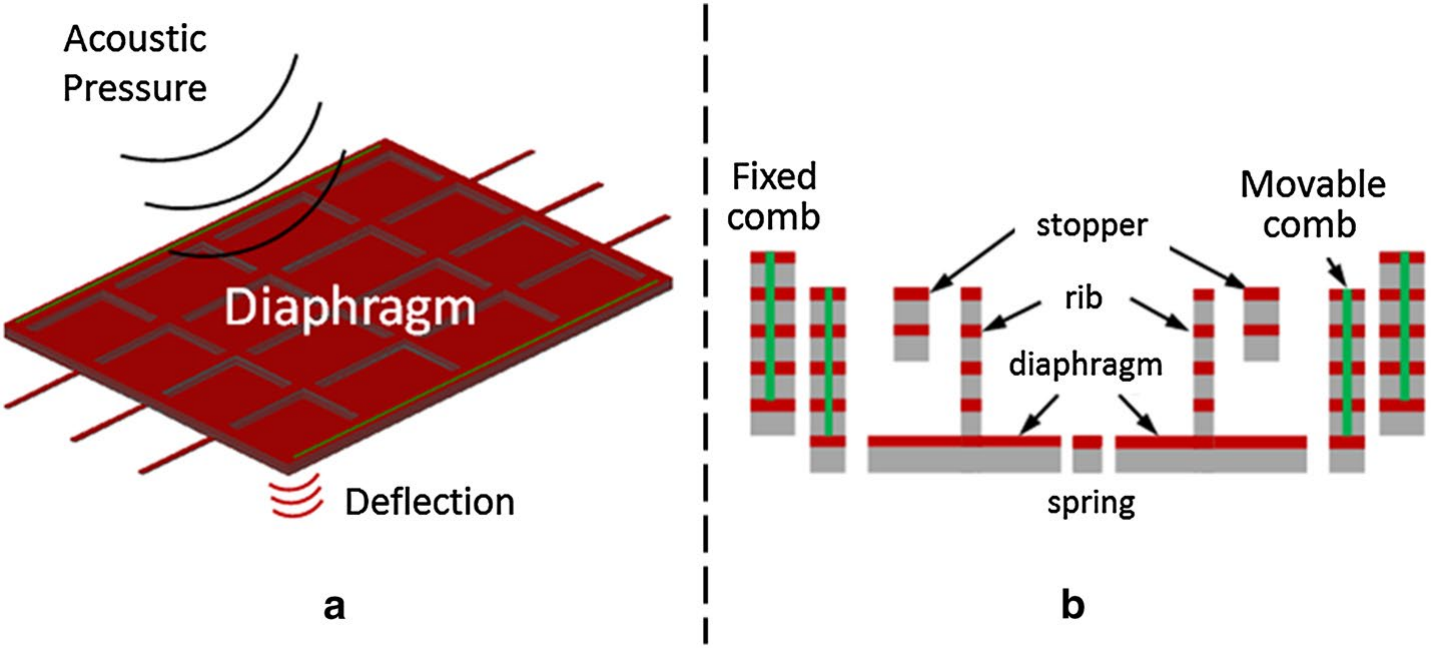
**a** Illustration of the working principle of the MEMS microphone, **b** cross sectional diagram of the capacitive MEMS microphone.

The capacitive-type sensor based on the Micro-electro-mechanical system (MEMS) technology detects acoustic pressure with a change in its nominal capacitance value [25–29]. Figure 5a shows the working principle of the capacitive MEMS sensor. The center piece which is the diaphragm is a suspended weight (proof mass) that is free to move. This proof mass is electrically isolated from a static fixed structure depicted by the fixed comb in Figure 5b, thus having a nominal capacitance value between them. When the diaphragm is subjected to an acoustic pressure source, it will start to move in harmony with the source thus causing changes in its nominal capacitance value. The capacitive MEMS sensor has the advantage of small size, mass production and better temperature stability. In addition, it is compatible with conventional complementary metal-oxide-semiconductor (CMOS) technology; hence, when combined with integrated circuit it makes it possible to develop high performance HS sensor system.

**Figure 6:**
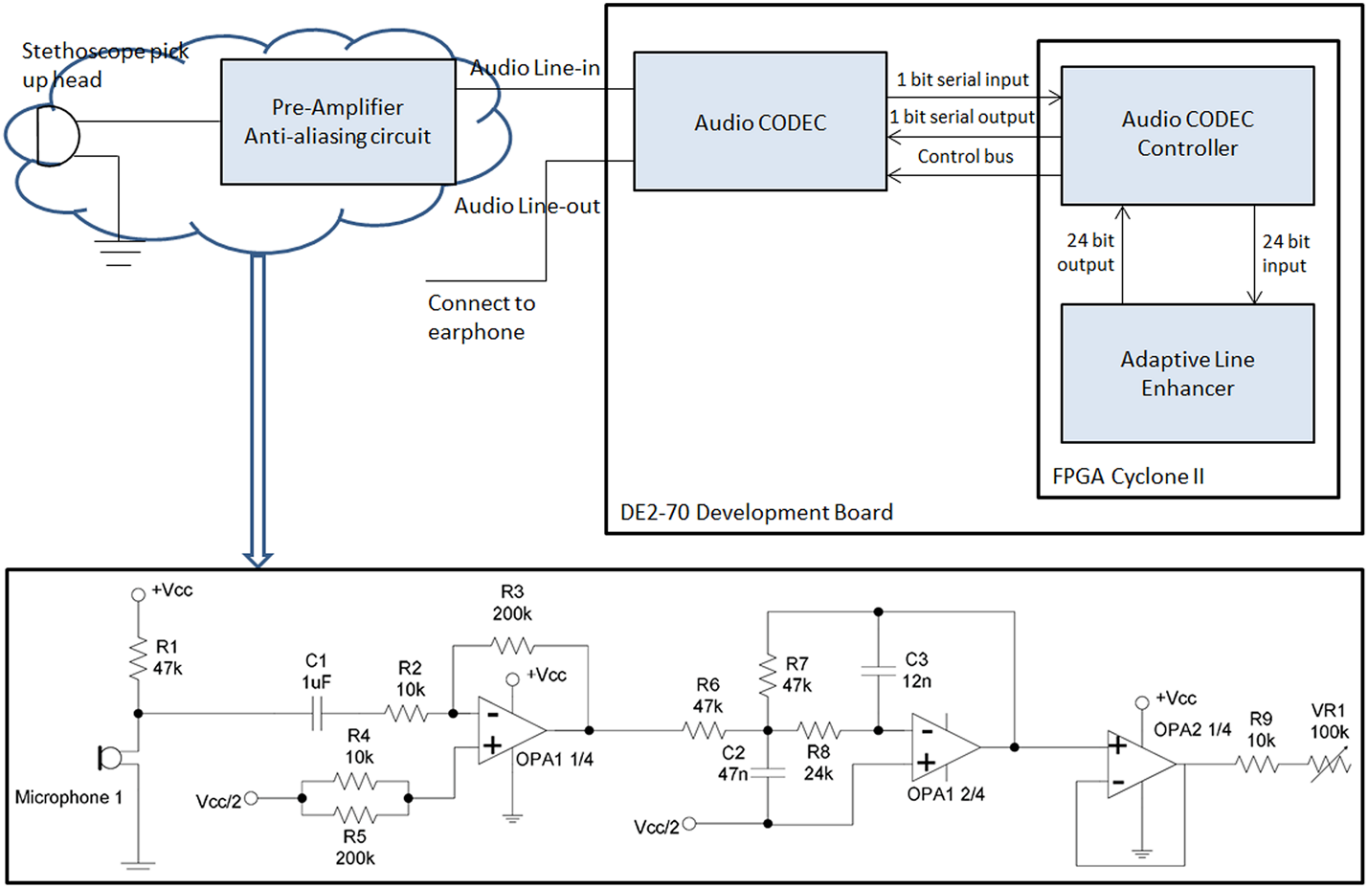
Sensor, pre-amplifier, anti-aliasing, audio CODEC and denoising in an electronic stethoscope [30].

The signal captured by the sensor of the electronic stethoscope is fed into the pre-amplifier and anti-aliasing circuit as discussed in earlier section. Figure 6 shows the two stages of the signal path from the stethoscope pickup with a microphone as the sensor [30]; therein, the signal from the microphone goes through an amplifier with a gain of 20, followed by an anti-aliasing low pass filter using OPA134 with a cut-off frequency at 2 kHz. The audio CODEC block and signal denoising block are also shown in Figure 6; the CODEC block is a single device that encodes analog sound signal as digital signals and decodes digital back into analog; the denoising block is used to suppress the unwanted noises. The detailed discussion on the HS denoising will be provided in the following subsection.

### Heart sound denoising

HSs are very often disturbed by various factors, which can prohibit their automated analysis. In general, the HS noise can be divided into two groups: external and internal [31]. The external disturbances include a wide frequency and intensity spectrum of noise caused by speech and noise caused by motion, whereas the group of signals with internal origin disturbances consists of mainly signals caused by digestive and respiratory processes. Moreover, there are many other types of noise, which during the measurement may occur occasionally, such as vocal (coughing, laughing), physiological (muscle movements, swallowing), sensor (rubbing) and ambient (knocking at the door, ambient music, phone ringing, footsteps) [32]. Due to the existence of such noises, some components of the HS may become extremely hard to hear during auscultation—especially the murmurs which have lower amplitude and similar characteristics to noise. Therefore, the development of precise noise removal algorithms, which are capable to work in noisy environments, is of great importance and is the research subject of many scientists.

A total of 21 articles for HS noise reduction techniques were selected for review studies, and the overview of nine (out of 21) articles is tabulated in Table 2. Generally speaking, the HS denoising algorithms can be categorized into two groups: denoising in time domain and transform domain.

**Table 2:** Heart sound denoising techniques

References	Category^a^	Sensor type	No. of sensors	Method	Outcome
[33]	Time	Electret condenser microphone	2	LMS-ANC	External noises are reduced by 12.92–16.11 dB
[34]	Time	Electronic stethoscope	1	Single input ANC	Most of breath sounds and noises are suppressed, performance robust to heart beat rate
[35]	Time	Microphone	1	LMS-ALE, RLS-ALE	HS recovery and noise reduction are 91–93% and 89–92%, respectively, for LMS-ALE; for RLS-ALE, they are 95–97% and 90–92%
[36]	Time	NA	NA	Variable step size Griffith LMS-ALE	The SNR gain is 7.85 dB
[37]	Frequency	Audicor system [38]	2	SS, FFT	Better noise removal; improved ability to detect S3 and S4 sounds
[39]	Frequency	Electronic stethoscope with electret microphone	1	DWT, Hilbert transform	Recommendations on optimal parameters selection are given
[40]	Frequency	Microphone	1	DWT, LMS-ALE, RLS-ALE	RLS-ALE and DWT with SURE shrinkage give best results
[41]	Frequency	NM	NM	EMD	EEMD seems to be a good solution for HS denoising
[42]	Frequency	NM	NM	EMD	S1 and S2 can be extracted with high accuracy

*NA* not available, *NM* not mentioned.

^a^Category: denoising in time/frequency domain.

**Figure 7:**
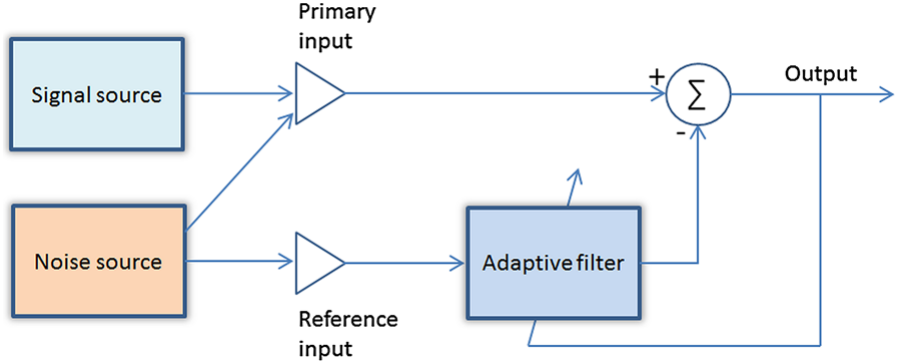
The structure of adaptive noise canceller.

*Time domain denoising* An adaptive noise canceller (ANC) is employed for external noise reduction [33, 43, 44]. The ANC, as shown in Figure 7, consists of two sound transducers. The first one, called primary input, is used to pick up the noise corrupted HS. The second one, named reference input, is used to only pick up the environmental noise correlated in some unknown way with the primary noise. The reference input is filtered and subtracted from the primary input to obtain the signal estimate.

Since both HS and environmental noise are time-varying, the filter uses an adaptive algorithm to change the value of the filter coefficients, so that it acquires a better approximation of the signal after each iteration. Some of the most popular of such adaptive algorithms are least mean square (LMS) algorithm, normalized LMS (NLMS) algorithm, subband LMS (S-LMS) algorithm, subband NLMS (S-NLMS) algorithm and recursive least square (RLS) algorithm [43]. To further enhance the HS, a type I Chebyshev infinite impulse response (IIR) band pass filter was also used in [44] to filter out the HF and LF bands, and only keep the major frequency band of interest.

One major drawback of the ANC is that it needs two input channels (e.g., two microphone sensors) as a primary signal and a reference signal. However, a single stethoscope can provide just one input sound. To overcome this issue, the authors in [34] have proposed a single input ANC (SIANC) for suppression of breath sound in a cardiac auscultation sound. The proposed SIANC uses a reference generation system which generates a reference signal using the primary signal. The reference generation system consists of a HS detector, a control unit and a reference generator. The HS detector is used to detect the presence of major HS components, i.e., S1 and S2. When S1 or S2 is detected, the reference signal is taken from the reference generator output, and hence the main HS components are excluded from the reference signal. However, the SIANC is only suitable for normal HS denoising. Its performance will be largely degraded if the original HS comes from a patient with certain heart disease, where murmur signal exists between S1 and S2. The ultimate objective of any ANC is to only suppress the external noise not produced by the heart. With SIANC, the murmur signal can be easily transferred to the reference generator and subsequently taken as part of the reference signal, resulting in the elimination of murmur from the final output HS.

**Figure 8:**
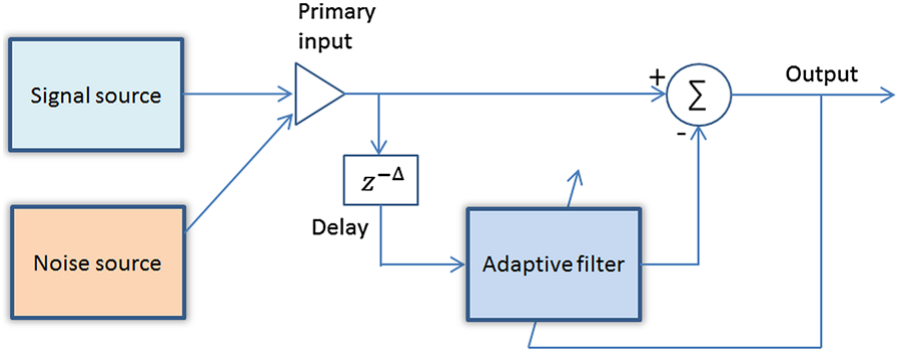
The structure of adaptive line enhancer.

Apart from ANC, the adaptive line enhancer (ALE) is also used for denoising the HS. The main advantage of it is that, unlike ANC, it does not require any reference signal to eliminate the noise signal, and thus the additional sensor becomes unnecessary. As seen from Figure 8, the reference signal in ALE is the delayed version of the primary signal. In [35], two types of ALE were implemented, namely LMS-ALE and RLS-ALE. The study shows that RLS-ALE is a method superior to the LMS-ALE for HS denoising. The former is found to be more consistent and accurate though computationally expensive.

One challenge with the conventional LMS and NLMS is the proper choice of step size, since there is a trade-off between the convergence rate and the misadjustment error which is the ratio of excess error to the optimum error [45]. A larger step size can be used to speed up the convergence, but this will increase the misadjustment error. To overcome this issue, the authors in [36] proposed to implement the ALE with variable step-size Griffith LMS. This method not only facilitates faster convergence, but also smaller convergence error/misadjustment.

*Transform domain denoising* The signal transform is defined as converting the signal from one domain (normally time domain) to another domain, with the purpose of extracting the characteristic information embedded within the time series that is otherwise not readily observable in its original form. The Fourier analysis forms the basis, as the most popular transformation technique to find the frequency content of a signal. The Fourier transform (FT) reveals the frequency composition of a time series by transforming it from the time domain into the frequency domain. The FT is usually implemented in the form of the fast FT algorithm (FFT). One drawback of FT is that it is not suited for analyzing non-stationary signals because it does not reveal how the signal’s frequency contents vary with time. On the other hand, as most real world signals are generally non-stationary in nature (such as HS signals), a new signal processing technique that is able to handle the non-stationarity of a signal is needed. The short-time FT (STFT) is one solution to overcome the limitations of the FT [46]. The STFT analyzes a small section of the (stationary or non-stationary) signal at a time, which is known as windowing. The time domain signal is decomposed into a 2D time–frequency representation using STFT, and the variations of the frequency content of that signal within the window function are revealed. The problem with STFT is a compromise in resolution. A wide window gives better frequency resolution but poor time resolution. A narrow window results in good time resolution but poor frequency resolution. This resolution problem of STFT is solved by wavelet transform (WT) [47] which uses variable-length window. It was developed as a method to obtain high resolution in both time and frequency. A WT in which the wavelets are discretely sampled is known as the discrete WT (DWT).

One type of HS denoising algorithm using FFT and STFT is called the spectral subtraction (SS) based methods [48]. In this method, an average signal spectrum and average noise spectrum are estimated in parts of the recording and subtracted from each other, so that average SNR is improved. One drawback of the classical SS is that it often distorts the signal and introduces certain artifacts called “musical noise”. In this regard, Ephraim and Malah [49] have proposed a minimum mean-square-error (MMSE) based short-time spectral amplitude (STSA) estimator that is closely related to SS. This technique uses a “decision-directed” approach to estimate the SNR on-line in the spectral domain. The SNR is then used to determine a Wiener filter gain applied to the spectral amplitudes for estimating the signal. A number of improved algorithms based on the MMSE–STSA have been proposed for HS denoising application [37, 50]. For stationary noises, these methods can achieve a high amount of noise suppression; however, the amount of non-stationary noise suppression tends to be dependent on the speed with which the noise spectrum is updated.

The most widely used noise reduction methods of HS are wavelet denoising algorithms which are based on DWT [39, 40, 51–58]. The general wavelet denoising procedure is as follows:

- *Decomposition* Apply DWT to the noisy signal to produce the multi-level wavelet coefficients. The WT will compress the energy in a signal into few large components, whereas the noise is disorderly and characterized by small coefficients scattered throughout the transform.
- *Thresholding* Select appropriate threshold value at each level, and threshold method to neglect the smaller coefficients from the wavelet-decomposed details.
- *Reconstruction* Apply inverse DWT to the resultant wavelet coefficients to obtain a denoised signal.

One assumption made in [39] is that the environmental noise can be modeled as white Gaussian noise. However, according to the authors of [51], some color noises can also be found in the real HS signals collected by using their self-produced HS recording device. More specifically, based on the study of signal spectrum, the power spectral densities (PSD) of the noise registered during real HS signal measurements is very similar to the PSD of red or pink noise. Therefore, some investigations on the DWT parameters selection have been done in [51] with the noise characterized and modeled as colored. And it is concluded that the best results of wavelet denoising are obtained by the use of wavelet Coiflet 5 at the 10 decomposition level, minimax thresholding algorithm, and multiple level rescaling function.

Some shortcomings of DWT are that it has the shift variant property, and the wavelet coefficients are oscillatory in nature [52]. A small shift in the input signal may completely change the wavelet coefficient oscillation pattern around singularities. On the other hand, due to the non-ideal wavelet filter, some redundant frequencies will still be left after being firstly filtered by a wavelet filter [53, 54]. Hence, a biomedical signal denoising algorithm based on the dual-tree complex wavelet transform was presented in [52], with the important additional properties including an anti-aliasing effect and nearly shift invariant. Experiment results show the effectiveness of the method in terms of SNR improvements.

The performance of DWT with Stein’s Unbiased Risk Estimate (SURE) shrinkage and Bayes shrinkage were studied and compared to that of LMS-ALE and RLS-ALE [40]. It is evident from the analysis that RLS-ALE and DWT with SURE shrinkage give the best results with HS recovery of 95–98% and noise reduction of 88–92%.

It was mentioned earlier that the adoption of ANC algorithm usually requires two sensors, whereas only 1 input channel is needed for DWT thanks to the un-necessity of reference signal input. However, a novel DWT based method which allows a more effective cancellation of noises was proposed [31], by which significant improvement of signal quality can be achieved at the cost of one additional microphone sensor. Two identical high-sensitivity electret microphones are employed in the system, wherein the first microphone records the signals of heart, internal noises as well as external noises; the second microphone measures the external noises. The internal noises and external noises are suppressed in a two-step sequential processing with wavelet decomposition done on both the recorded signal channels.

The efficiency of wavelet based denoising techniques greatly depends on the choice of threshold parameter. A very large threshold cuts too many coefficients, resulting in some useful signal loss. On the other hand, a too small threshold value allows many coefficients to be included in reconstruction whereby excess noise will be retained. Besides the categorization of thresholding techniques as hard thresholding and soft thresholding, the threshold choosing methods can also be divided into two types according to their wavelet level dependence: global thresholding and level-dependent thresholding. The former chooses a single value of threshold to be applied globally to all empirical wavelet coefficients, while the latter selects different thresholds for each wavelet level. Some improved global thresholding function in wavelet domain can be found in [55, 56], and the wavelet subband dependent thresholding functions were proposed in [57, 58] with encouraging results reported.

Besides WT, the empirical mode decomposition (EMD) [59], as another method for denoising, has become rather popular. The EMD process is a way to decompose a signal into so-called intrinsic mode functions (IMF), and obtain instantaneous frequency data. It has proven to be quite versatile in a broad range of applications for extracting signals from data generated in noisy non-linear and non-stationary processes. Mode mixing is one of the drawbacks of the original EMD, wherein a single IMF may consist of signals of widely disparate scales or a signal of similar scales may reside in different IMFs. In order to overcome this problem, the Ensemble EMD (EEMD) constitutes a noise-assisted data approach proposed in [60]. This algorithm defines IMF components as an ensemble of trials, each consisting of original signals contaminated with white noise of finite amplitude. A number of denoising methods of HS using EMD or EEMD can be found in [41, 42]. It is concluded that the EEMD seems to be a good solution for HS denoising, provided the useful subtle features have been detected previously [41].

Another two recently proposed HS denoising methods [61, 62] are also worth further exploration and investigation on their effectiveness with more real noisy HS data, especially abnormal HS data. A denoising and segmentation technique for the second HS S2 using matching pursuit (MP) was presented earlier [61]. The MP is an algorithm used to decompose a signal into a linear expansion of waveforms that are selected from a dictionary of time–frequency functions (called atoms) [63]. MP can be applied for denoising, analysis and synthesis of the HS. One drawback of MP based algorithm is the lack of explicit models. This has motivated the authors in [62] to develop a new dynamical model for HS, which is capable of synthesizing realistic HS signals. A new HS denoising framework using extended Kalman smoother was established subsequently based on the proposed HS dynamical model. Simulation results demonstrate the superior performance compared to that of WT denoising methods [62]. Furthermore, this framework may also be helpful for joint ECG–PCG real-time processing for clinical application. However, the major limitation of the method is that the dynamical model was built to only model the main HS components, i.e., S1 and S2. Hence performance degradation may be encountered if other components of the HS also exist.

It was mentioned earlier in this section that the lung sound (LS) constitutes one component of the HS noise. The LS is produced by the structures of the lungs during breathing. In fact, even though most of the denoising techniques discussed above are claimed to be able to suppress the LS from the HS, analyzing the nature of the LS and how they affect HS often requires another independent study. Among all the solutions, blind source separation or specifically the independent component analysis (ICA) techniques are most widely adopted. The basic idea of ICA is to find a demixing matrix to extract out the desired signal (the HS) from the linearly mixing signal (the HS and LS). The details can be found in [64, 65] and the references therein.

### Heart sound segmentation

Signal segmentation is usually carried out after denoising stage. The simplest way of segmentation is through the use of sliding window [66]. This technique simply partitions the whole HS data into a number of segments with the same duration, without considering the positions of starting point and ending point for each segment. The more commonly adopted segmentation technique in HS analysis is performed on the basis of the cardiac cycle, since in most cases the activities in the HS signal relating to given disease are contained in a single interval of cardiac cycle. Specifically, the segmentation algorithm divides the HS data into portions, each of which consists of four parts: S1, the systole, S2 and the diastole.

The majority of the attempts in segmenting the HSs use the ECG signal and/or carotid pulse as the reference signal [67–71]. This method, often called indirect segmentation, demarcates the HS boundaries using the QRS complex and T-waves of ECG signal. The disadvantage of indirect segmentation is that, apart from the employment of ECG electrodes, the complete segmentation may become difficult because the T-wave is too weak to be identified in some patients [72]. Another disadvantage of using ECG as a reference is that the timing between electrical and mechanical activities in a cardiac cycle is not constant for all patients, because of a variety of pathological conditions [73].

**Figure 9:**
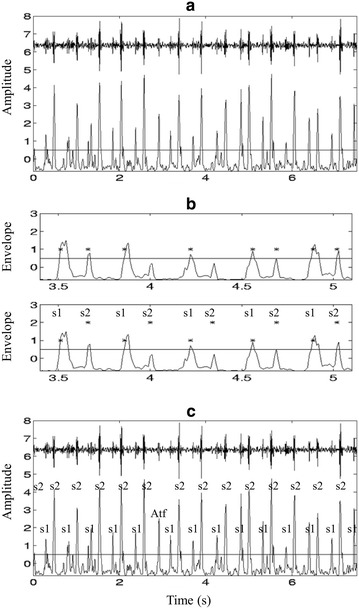
Envelogram based heart sound segmentation **a** original signal and its average Shannon energy, **b** recovering the “lost” peaks, and **c** identified S1 and S2 and artifacts.

The direct segmentation methods are well established in the literature, where the cardiac cycle boundary is located based on the HS signal itself without a reference to the ECG. The most famous method of direct segmentation is the envelogram based approach presented in [74] and illustrated in Figure 9, where the following four steps are performed:

- Calculate the envelope of the HS, using normalized average Shannon energy; Figure 9a shows the original HS signal and its normalized average Shannon energy.
- Detect the peaks of the envelope with a threshold. Several additions have been made in this procedure to reduce the peak detection errors, such as rejecting extra peaks and recovering “lost” peaks. The "lost" peaks are recovered by lowering the threshold (Figure 9b).
- Establish the one-to-one correspondence between the peaks and the S1 and S2, based on the fact that the largest interval of a recording is the diastolic period, as depicted in Figure 9c.
- Form the cardiac cycle using the S1–S1 intervals.

The envelogram based method in [74] was extended in [75] such that, instead of performing the four-steps processing on the original HS data, a DWT was carried out and some wavelet coefficients were selected to cue the same segmentation procedure. The advantage of this extension is the lower sensitivity to ambient noises and recording locations. The basic theory behind the methods in [74, 75] is that a transform (Shannon energy and DWT) is performed to take the raw time domain HS data into another domain where S1 and S2 are emphasized. Other types of such transformation have been reported over the years for HS segmentation application, to name a few, STFT [76], PSD [77], homomorphic filtering, K-means clustering [78], MP [79], and EMD [80].

### Feature extraction and classification of heart sound

The feature extraction and classification of HS are the two key stages for automatic HS interpretation and diagnosis of heart dysfunction. A total of 53 articles were selected for review studies and the overview of nine (out of 53) articles is tabulated in Table 3.

**Table 3:** Heart sound classification techniques

References	Classification method		Normal HS	AR	AS	MR	MS
[81]	Markov Blanket Random Forests (multiple binary classification)	Analyzed sound/disorder	Y	Y	Y	Y	Y
		No. of cases	38	38	41	43	38
		Sensitivity	Accuracy of 90.22% for distinguishing between normal and abnormal; accuracies of 92.45 and 90.34% for distinguishing between AR-MS and AS-MR				
		Specificity					
[82]	ANN (multi-class classification)	Analyzed sound/disorder	Y	Y	Y	Y	–
		No. of cases	31	26	28	26	–
		Sensitivity	–	89.7 ± 5.9%			–
		Specificity	–	–	–	–	–
[83]	SVM (multiple binary classification)	Analyzed sound/disorder	Y	Y	Y	Y	–
		No. of cases	15	51 in total			
		Sensitivity	–	95.37%	94.02%	95.20%	–
		Specificity	–	95.49%	96.68%	96.33%	–
[84]	ANN, GMM, SVM (multiple binary classification)	Analyzed sound/disorder	Y	Y	Y	Y	–
		No. of cases	15	51 in total			
		Sensitivity	–	93.20%	92.63%	96.88%	–
		Specificity	–	95.87%	95.80%	92.78%	–
[85]	HMM, SVM (multi-class classification)	Analyzed sound/disorder	Y	Y	Y	Y	Y
		No. of cases	80	6	9	9	12
		Sensitivity	–	67%	67%	67%	83%
		Specificity	99%	–	–	–	–
[86]	ANN (multi-class classification)	Analyzed sound/disorder	Y	Y	Y	Y	Y
		No. of cases	4	4	4	4	4
		Sensitivity	–	100%	98%	100%	100%
		Specificity	100%	–	–	–	–
[87]	SVM (binary hierarchical classification)	Analyzed sound/disorder	Y	–	Y	–	Y
		No. of cases	225	–	60	–	60
		Sensitivity	–	–	85%	–	95%
		Specificity	98.67%	–	–	–	–
[88]	SVM (multiple binary classification)	Analyzed sound/disorder	Y	Y	Y	Y	Y
		No. of cases	38	38	41	43	38
		Sensitivity	Accuracy of 91.43% for distinguishing between normal and abnormal; accuracies of 92.11 and 91.67% for distinguishing between AR-MS and AS-MR				
		Specificity					
[89]	SVM (binary hierarchical classification)	Analyzed sound/disorder	Y	Y	Y	Y	Y
		No. of cases	6	6	4	6	9
		Sensitivity	–	93.75%	92.31%	96.97%	90.63%
		Specificity	99.49%	–	–	–	–

*Y* yes.

*Instrumentation for heart sound recording* The sensors that are used most often in HS classification are electronic stethoscope with microphone and piezoelectric sensor, which can acquire a wide range of frequencies between 0 and 2,000 Hz. Few notable recording instruments used by earlier researchers are electronic stethoscopes from Welch–Allyn (piezoelectric sensor), HP (piezoelectric sensor), 3 M (piezoelectric sensor) and Cardionics (microphone). More details on the commercially available HS recording instruments will be provided later in the market review.

*Analyzed heart sounds/disorders* Cardiac dysfunction is diagnosed through the detection and analysis of abnormal HSs, such as S3, S4 and most importantly the murmurs. In the section of “Characteristics of heart sounds”, we listed the 12 common murmurs. In some previous research work done by the researchers [90–104], the HS signals are analyzed and classified as normal HS and murmur, based on which the HS data are simply categorized as normal and abnormal. With more sophisticated algorithms, the classifiers can even distinguish different types of murmurs. The four distinct classes of murmur that are most commonly considered by the classifiers are MS, mitral regurgitation (MR), aortic stenosis (AS) and aortic regurgitation (AR) (e.g., [81, 82, 105–109]).

*Test heart sound data* Various normal and abnormal HS data collected from public HS database were used for algorithm validation in 17 out of the 53 selected studies. For most of the rest studies (apart from ten studies in which the type of test data was not mentioned), real HS data recorded from several to dozens of subjects (normal subjects and patients) were used to test the effectiveness of the algorithms proposed therein. Here one should note the differences between the “number of HSs” and “number of subjects” that are commonly mentioned in the literature. The number of HSs is usually much larger than the number of subjects, since multiple pieces of HS may come from single subject recording at different time or different chest locations.

*Methods for feature extraction* The extraction of features, which is the process of identifying distinctive properties from a signal, plays a major role in the effective classification of HS. The features can be extracted from the signals in one of the four domains: time domain, frequency domain, statistical domain and time–frequency domain. Some typical feature extraction techniques used in computer-based HS analysis are:

- Time domain: timing, intensity, frequency location over time and shape [83], zero crossing rate, transition ratio [84], power, instantaneous energy [110], systole duration, diastole duration [111], durations of S1 and S2 [112], etc.
- Frequency domain: spectral power based features [84, 113], instantaneous frequency [110], dominant maximum frequency [111, 114], etc.
- Statistical domain: skewness, kurtosis, chaos [84], entropy [90, 105], heart rate [96, 111], etc.
- Time–frequency domain: mel frequency cepstral coefficients (MFCC) [85, 100, 101, 115–117], DWT [67, 86, 92, 93, 97, 102, 106, 118–128], STFT [128, 129], EMD [87], etc.

**Figure 10:**
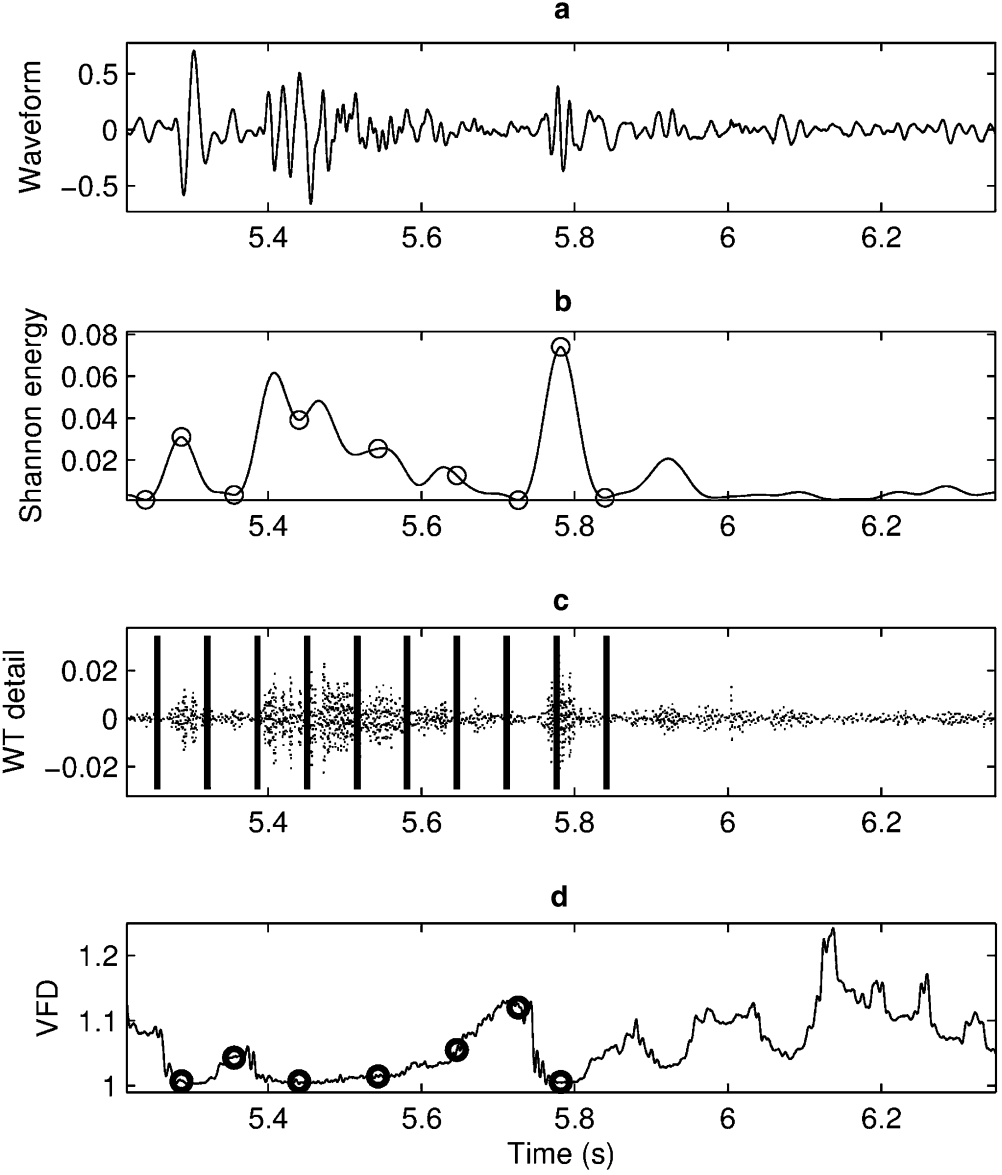
An example of feature extraction in [132]: **a** original HS waveform, **b** time domain: signal’s envelop extracted from Shannon energy, selected features indicated by *rings*, **c** time–frequency domain: wavelet detail, absolute sum between time markers (*vertical lines*) forms feature values, and **d** statistical domain: variance fractal dimension, selected features indicated by *rings*.

The MFCC and DWT based features are most widely used for HS classifications, and the results presented recently in the literature have demonstrated their effectiveness. In fact, to achieve the best classification performance, some mixtures of features from different domains are always employed. A set of optimal features extracted from time, frequency and statistical domain was introduced in [130]. In [131], the whole feature set was formed by a total of 18 time domain features, 9 DWT features and five entropy features. By using the method presented in [88], a feature vector of dimension 100 can be created with four from the statistical domain, 88 from the morphological domain, and eight belonging to the frequency domain. A large number of features were extracted in [132] and they are depicted in Figure 10, showing a combination of three features from time domain (Shannon energy), time–frequency domain (wavelet detail), and statistical domain (variance fractal dimension).

*Methods for classification* HS classification is a challenging task due to the nature of non-stationary property of the HS signal and large variations for the HS signals belonging to the same category. According to the review results, the artificial neural network (ANN) and support vector machine (SVM) algorithms are the classification techniques that are mostly used. The accuracy provided in [86] is 99% in classifying normal HS, MR, MS, pulmonary stenosis (PS), AR and summation gallop (SG) using ANN. The recognition rate reported in [87] was 95.56% using SVM in classification of normal HS, MS, AS and ventricular septal defect (VSD). The ANN has the ability to adapt well with complex non-linear data (such as HS) and classify it accurately and effectively. SVM, which is a new classification technique, has been used as a classification tool with a great deal of success in various applications areas. Other methods that have been used in the classification of HSs are Gaussian mixture model (GMM), hidden Markov model (HMM), k-nearest neighbors (KNN), decision trees and Bayesian networks, etc. Interested readers can refer to [133] and the references therein, for the detailed discussions and comparisons of different classification techniques.

**Figure 11:**
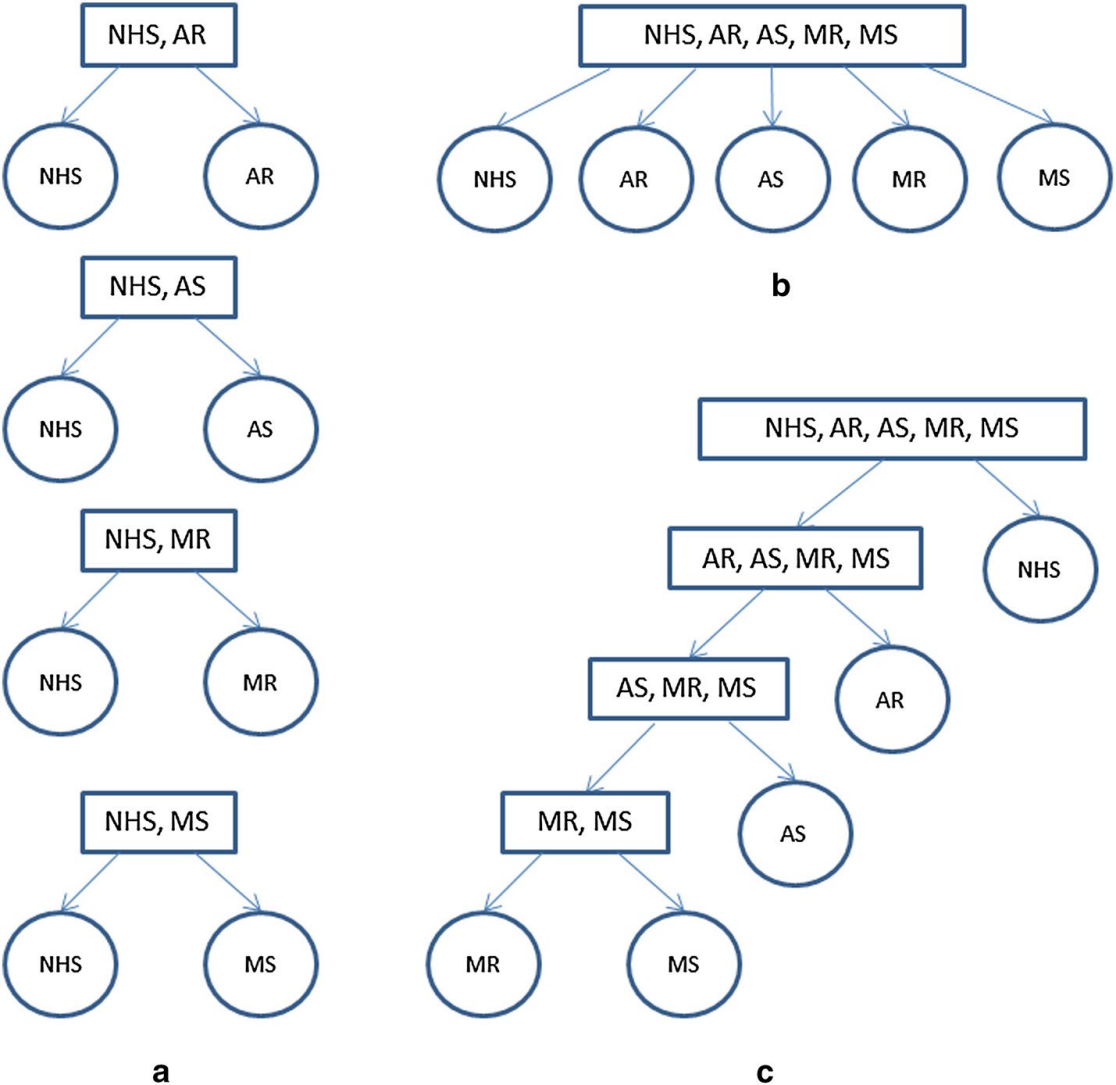
**a** Multiple binary independent classifications, **b** Multi-class classification, and **c** Binary hierarchical classification. *NHS* normal heart sound.

The structure of the classifier depends on the goal of the system which may be either to screen normal from abnormal HS or to identify a specific heart disease. Figure 11 depicts three approaches to conduct HS classification, assuming the normal HS (NHS), AR, AS, MR and MS are those to be classified. The objective of multiple independent binary classifications (Figure 11a) is to categorize each of the heart disorder against NHS or a different heart disorder [81, 83, 84, 88]. The more commonly adopted approaches are multi-class classification (Figure 11b) [82, 85, 86] and binary hierarchical classification (Figure 11c) [87, 89], where the former classifies the HS instances into one of more than two classes; and the latter distinguishes one class from the remaining classes at each hierarchy level.

Proper validation of a classification method is important to see its effectiveness. Usually, the available data are divided into training set and testing set, e.g., in [87], 90% of the HSs (both normal and abnormal) were applied as training samples and the remaining 10% as testing samples; in [99], 70% of the HSs were randomly selected for training while 30% were taken for testing. Two commonly selected validation techniques are “repeated k-fold cross validation” [81, 84, 91, 94, 98, 99, 117, 128], and “leave-one-out” method [85, 88, 107].

Table 3 lists the summaries of classification performances in terms of sensitivity and specificity for nine selected articles, and the following observations can be made (here only AR, AS, MR and MS are considered since they are the most commonly diagnosed heart disorders):

- 8, 9, 8 and 6 out of the nine selected articles considered the diagnosis of AR, AS, MR and MS, respectively.
- The mean (sensitivities, specificities) for diagnosing AR, AS, MR and MS are (89.8, 98.0%), (88.4, 98.3%), (91.0, 97.52%) and (92.2, 99.29%).

The good classification performance obtained suggests that these techniques are potentially useful for medical application, even though it is still premature to look at their real diagnostic value as will be discussed in later sections.

Extreme learning machine (ELM), as another new machine learning method, has attracted extensive attention recently due to its remarkable advantages such as fast operation, straightforward solution and strong generalization [134]. However, the use of ELM in HS analysis is found to be very limited in the literature. The diagnostic potential in this domain of ELM has definitely not been sufficiently explored as yet, and so further research is required in this direction.

## Market (state-of-the-art) products review results

The review results on the state-of-the-art products are provided in this section, validating the needs, problems and gaps from the medical and market point of view as well as from a technology push perspective with an overview tabulated in Table 4.

**Table 4:** Summarized results from the market review

References	Product Name	Feature highlights and characteristics	Sensor type	Algorithm
[135]	3 M Littmann Range	Bell mode (20–200 Hz) Diaphragm mode (100–500 Hz) Extended mode(20–1,000 Hz) Amplify sounds up to 24 times	Piezoelectric sensor	Ambient noise reduction using ANC Friction noise dampening No built-in signal classifier
[136]	Thinklabs One Digital	Filter for adjusting sound in bell/diaphragm mode, and acoustic mode Amplify sounds up to 100 times	Patented capacitive transducer	Ambient noise reduction, specific HSs extraction (e.g., valve clicks) and LS removal by filtering No built-in signal classifier
[137]	Welch-Allyn Elite Electronic Stethoscope	Bell mode (heart): 20–420 Hz Diaphragm mode (lungs) 350–1,900 Hz Integrated ECG	Piezoelectric sensor	Ambient noise reduction with novel sensor design No built-in signal classifier
[138]	Cardionics E-scope II	Selectable frequency response for HS and LS. Amplify sounds up to 30 times Complement system enabling ECG capability	Microphone	NM
[139]	Rijuven CardioSleeve	Stethoscope add-on device Allows regular stethoscope to have simultaneous acquisition and quantitative measurements of ECG and cardiac acoustical data	NA, stethoscope add-on device	Normal and abnormal HSs identification and quantification Murmur detection
[140]	Ekoscope	Bell mode Diaphragm mode Diaphragm plus mode Eight built-in EKG electrodes	NM	NM
[16]	JABES Life Sound System	Diaphragm mode: 200–500 Hz Bell mode: 20–200 Hz Wide mode: 20–1,000 Hz Amplify body sounds up to 20 times	NM	Heart beat calculation Ambient noise and hand tremor cancellation No built-in signal classifier
[141]	ViScope	Bell mode Diaphragm mode Body sound amplification up to 30 times Real-time visual display of multiple waveforms	NM	Patented visualization algorithms

*NA* not available, *NM* not mentioned.

Development of the electronic stethoscope is gaining an edge over traditional stethoscope mainly due to the advanced sensor technologies, digital signal processing techniques as well as the digital sound transmission capabilities of digital stethoscopes. Most stethoscope manufacturers are focusing on developing the devices with enhanced acoustics, better performance and innovative designs. One example of the state-of-the-art electronic stethoscope has been given in Figure 1.

Unlike acoustic stethoscopes, which are all based on the same physics, transducers in electronic stethoscopes vary widely:

- The simplest and least effective method of sound detection is achieved by placing a microphone in the chest piece. This method suffers from ambient noise interference and has fallen out of favor.
- Another method, used in Welch–Allyn’s Meditron stethoscope, comprises placement of a piezoelectric crystal at the head of a metal shaft, the bottom of the shaft making contact with a diaphragm.
- 3 M also uses a piezoelectric crystal placed within a foam behind a thick rubber-like diaphragm.
- Thinklabs’ Rhythm 32 inventor, Clive Smith uses an electromagnetic diaphragm with a conductive inner surface to form a capacitive sensor. This diaphragm responds to sound waves identically to a conventional acoustic stethoscope, with changes in an electric field replacing changes in air pressure. This preserves the sound of an acoustic stethoscope with the benefits of amplification.

Almost all electronic stethoscopes in the market are equipped with configurable filters with different frequency response modes for listening to the heart, lungs and even other human body sounds. These band pass filters with user-selectable passband frequencies are easy to implement and cost-effective. Besides the basic filtering mechanism, novel sensor design and noise reduction algorithm are also implemented in the products from 3 M, Thinklabs, Welch–Allyn and JABES to suppress the ambient noise, as well as the friction noise due to either patient’s body motion or physician’s hand tremor.

**Figure 12:**
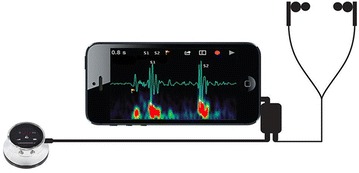
Electronic stethoscope with mobile phone.

Currently this is no product offering for a truly specialized stethoscope tailored to the automatic heart disease diagnosis. Most of the products offer a wide range of generalist features for general auscultations. Nevertheless, thanks to the signal recording and transmitting capability offered by these products, the digital HS data can be recorded locally and transferred to a PC for visualization and further analysis. Some electronic stethoscopes can become portable by establishing connections to other handheld devices like mobile phone or wirelessly transmitting the sound signals to a remote processing unit through Bluetooth. Figure 12 shows an electronic stethoscope with a mobile phone [136] to view the HSs and send the sound recordings instantly for telemedicine applications.

Rijuven is a new entrant providing a “generalist” stethoscope which also encompasses the S3, S4 and murmur sound detection. The CardioSleeve stethoscope attachment enhances a stethoscope by providing synchronized digital auscultation with ECG. With acoustic HS and ECG data, the analyzing software implemented in a remote device can be utilized to visualize the HS and detect murmur and cardiac dysfunction.

## Smartphone stethoscope apps review results

With the rapid development of smartphone technology, mobile health (mHealth) can support daily practice for health services and information. The mHealth applications include the use of mobile devices in collecting clinical health data, delivery of healthcare information to practitioners, researchers and patients, real-time monitoring of patient vital signs and direct provision of care (via mobile telemedicine) [142]. This section provides a brief review summary on the newly emerged smartphone stethoscope apps. The summaries of five typical stethoscope apps are provided in Table 5.

**Table 5:** Summarized results from the smartphone stethoscope apps review

References	Apps name	Feature highlights and characteristics	Algorithms	Outcome
[143]	SensiCardiac	Record: 15 s of HS recording at four locations Analyze: Instantaneous classification of HS Report: Keep record of cardiac assessment in the Cloud	HS segmentation Time/frequency features extraction HS signal classification using ANN	HS classified as either functional or pathological
[144]	StethoCloud	Cloud-based app that help diagnose pneumonia by listening to patient’s breathing sound Noise suppression and data analysis are done in a remote server (Cloud)	Noise suppression and “deep learning algorithm” (not specifically mentioned)	Diagnosis of pneumonia (and more respiratory conditions, e.g., asthma and heart failure in future)
[145]	Thinklabs	Record and display waveforms and spectrogram in real time Edit sounds on-screen Save, email recorded sounds Does not provide HS analysis and diagnosis	NM	Recorded sounds, waveforms and spectrogram
[146]	Mobile Stethoscope	It records body (heart, lung and bowel) sounds using built-in microphone of smartphone It does not need external stethoscope Record, save HS and display waveform Does not provide HS analysis and diagnosis	NM	Recorded sounds, waveforms
[147]	iStethoscope Pro	It amplifies and filters HS It uses built-in microphone of smartphone Record, save and email HS, display waveform Does not provide HS analysis and diagnosis	HS amplification with variable gain Low pass filtering with user selected cut-off frequency	Recorded sounds, waveforms and spectrogram

*NM* not mentioned.

**Figure 13:**
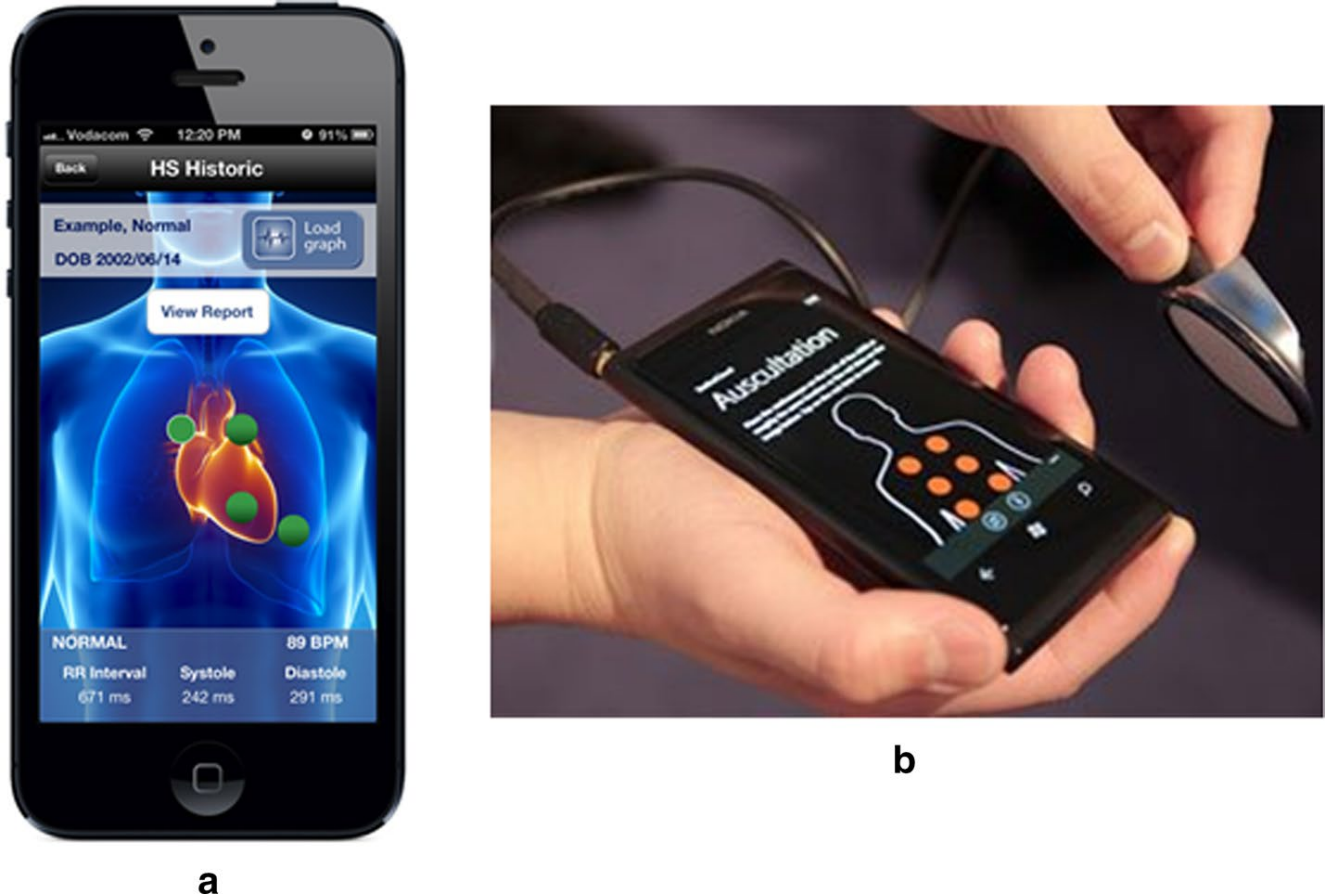
Smartphone stethoscope apps: **a** SensiCardiac, and **b** StethoCloud.

SensiCardiac [143], shown in Figure 13a, is declared as the world’s most accurate cardiac murmur assessment solution. Some of the key techniques involved in SensiCardiac are: HS identification, localization and segmentation, HS features extraction in time and frequency domain, and HS classification using ANN. The ANN in SensiCardiac was trained on a data set of nearly 2,000 HSs to objectively distinguish between pathological and innocent murmurs. Several independent clinical studies were conducted, and the results showed that a sensitivity and specificity of above 80% was achievable. It should be noted here that SensiCardiac is to be used as an aid to a physician in interpreting the data, and not intended as a sole means of diagnosis. StethoCloud [144], illustrated above in Figure 13b, is a cloud-based service that turns a Windows smartphone into a digital stethoscope. StethoCloud takes a relatively simple approach to replace stethoscopes and targets a common childhood killer. Different from SensiCardiac which performs the data analysis locally, both the noise suppression and data analysis for StethoCloud happen in the cloud using Windows Azure [148]. Thinklabs’s app [145] is one of the earliest developed medical stethoscope apps and it is used to record and visualize HSs on the smartphone.

All of the previous three apps require the smartphone to be connected with an external stethoscope, while some other stethoscope apps use the sensitive enough microphones embedded in the smartphone [146, 147]. Specifically, the microphone is placed on the key auscultation points. The app then enhances the sounds that the microphone hears on the smartphone; these sounds can then be recorded, saved and transmitted. Despite the advantages of non-necessity of external hardware, however, two main drawbacks exist for this type of stethoscope apps: firstly, the microphone is not easy to be placed in the exactly correct orientation and place, and secondly, the recorded sounds tend to be more prone to noise artifacts compared to those acquired using external advanced stethoscope.

## Discussions and recommendations

Since its invention 200 years ago, the stethoscope has been an invaluable bedside tool for auscultating HSs and over the years has undergone very few changes to both itself and the way in which it is used [149]. The stethoscope is employed by all doctors and nurses from primary to tertiary care. In the past decade, some fascinating debate has been sparked as to whether the stethoscope can be replaced by handheld ultrasound. Supporters of the handheld ultrasound-dominated view of future healthcare have pointed out that the use of ultrasound at the point of care can truly enhance patient care and outcomes by expediting diagnoses and treatment [150]. On the other hand, many others have advocated that handheld ultrasound devices be used as a sequel to or as an extension of the stethoscope, and not as a replacement for the stethoscope [151]. Stethoscopes have their primary use in cardiac diagnosis, which can be upgraded by electronic stethoscope. It is simply not possible for every nurse and doctor (and intern) to employ handheld echocardiography system universally, in place of the stethoscope. This is because handheld echocardiography requires highly trained personnel for data acquisition and interpretation [152]; also, costs will prohibit the exclusive use of handheld echocardiography systems [150]. Further at this stage, echocardiography cannot provide automated cardiac assessment, and requires extensive computerized analysis of heart function followed by development of indices for clinical diagnosis. So based on these considerations, there is a tremendous scope for the design of the very portably adoptable electronic stethoscope, so that each and every doctor and nurse can employ it for automated diagnosis in the clinical setting.

The emergence and further development of the computer-aided electronic stethoscope can become a big step ahead of the traditional acoustic stethoscope, by allowing physicians to hear and view, record and transfer the HS data, and obtain diagnostic assessment. With recent technological advancements on signal processing algorithms and machine learning techniques, the computer-aided diagnosis of cardiac dysfunction using electronic stethoscope will become a reality, and represent another tremendous step forward toward finding a truly acoustic based diagnostic tool in all medical clinics as well as in bedside use. The new intelligent computer-based stethoscope system can contribute to a new auscultatory semiology, based on reliable methods of signal analysis and automatic interpretation, and will be the preliminary point of care to be followed by other points of care modality like portable ultrasound. Hence, we are advocating that this new intelligent computer-based stethoscope system be made available for universal use in clinics and hospitals.

Generally, the ultimate goal of future research and development is to make the electronic stethoscope a truly diagnostic device, which interprets the HSs and offers diagnosis based on reliable and validated correspondence between HSs and cardiac and valvular disorders. To date, however, the computer based HS analysis has not yet been developed to a level that can be used for automated reliable diagnosis in the clinical setting. Almost all the available electronic stethoscopes and smartphone stethoscope apps in the market are only for general auscultation, i.e., hearing, recording and transferring the HS data. They are still far away from being called diagnostic devices.

In fact, the way that regulators would treat an electronic stethoscope depends critically on the claims made for it. Since the device would be used for diagnosis and treatment of disease, the electronic stethoscope would be treated as a medical device in US. The regulatory demands would be very different for:

- An “electrically amplified device used to project the sounds associated with the heart, arteries, and veins and other internal organs [153]”; most of the state-of-the-art products fall into this category [16, 135, 137, 140, 141].
- A device that interprets sounds and is to be used as an aid to a physician in interpreting the data; some devices of this nature already exist [139, 154, 155].
- A device that interprets sounds and offers diagnoses on its own, which would require extensive clinical data to get past the Food and Drug Administration (FDA).

The main factors limiting the electronic stethoscope to become a truly diagnostic device, in our opinion, are not only the diagnostic accuracy that the computer based system can provide, but more importantly the system’s reliability, robustness and transportability.

Firstly, the cardiac auscultations are usually performed in an environment which is not well controlled. The acoustic HS sensed by the stethoscope can be easily corrupted by the interferences and noises with versatile types and occurring at any time. The effectiveness of the computer based system can be largely affected by the existence of noises, due to the fact that the heart murmur signals has large frequency range and similar characteristics with background noises. The situation becomes even worse if the HS signals of interest are embedded in the high-amplitude noises. These problems can be eventually overcome by using HS recording sensors with high selective sensitivity and robust denoising algorithm, and these two solutions are part of the ongoing research trends in this field. As seen from the denoising algorithm review, the performance of existing noise reduction methods are evaluated mostly by using the clean HS signal from web HS databases combined with computer simulated noises; some are using clean real HS signal superimposed with simulated noises. The computer simulated noises, which are normally assumed as Gaussian distributed wide sense stationary, may largely deviate from the real noises that are usually encountered in the auscultation process. Therefore, more elegant noise suppression algorithms have to be developed and tested with various types of real interferences and noises.

Secondly, HS classification is a challenging task due to the nature of non-stationary property of the heart murmurs and large variations for the murmurs associating with the same heart disease. The characteristics of murmurs could vary for different patients with the same cardiac dysfunction, or even change for a single patient during different cardiac cycles. Hence, advanced feature extraction and classification techniques with robustness to the murmur variations are highly required to make the system more reliable. The number and choice of features is one of the most critical steps to the success of a classifier [156]. Selecting too little features will result in data under-fit, i.e., a lack of information to classify the more complex events. While at the other extreme, having too many features will lead to data over-fit, making the system have poor predictive performance, as it can exaggerate minor fluctuations in the data. As stated in [156], the number of features in the classifiers should be minimized and several approaches can be recommended to achieve this. Another important issue is to select independent training and testing data set for validation of the classification system. For HS analysis, different HS records from the same patient cannot be considered to be sufficiently independent, even if the records were from different chest positions or from different days [151]. Therefore, if repeated k-fold cross validation is adopted, it is not valid to simply divide all the HS segments into k folds. The proper procedure is as follows:

- Divide the enrolled subjects into k folds on an average.
- In each validation round, the HS segments associated with the subjects in one of the k folds are taken for validation, after the HS segments from the subjects in the remaining folds are used for training.
- Sometimes, the more reliable cross validation accuracy can be obtained by averaging the accuracy over multiple times of k rounds.

**Figure 14:**
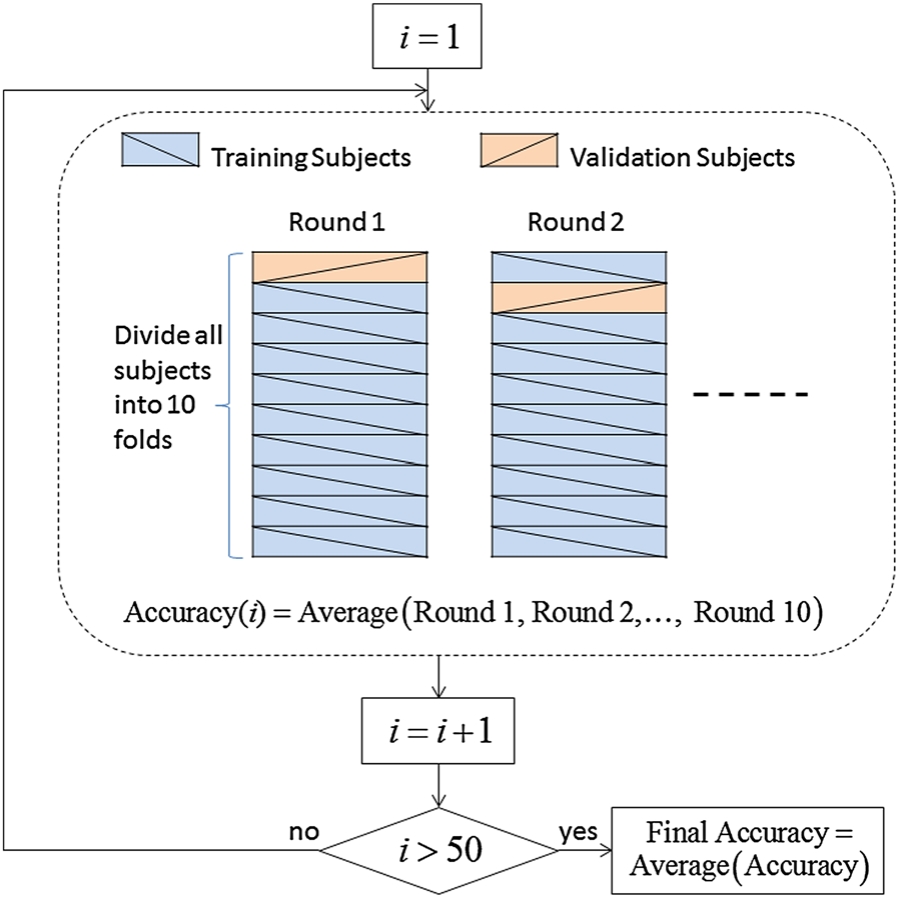
50 times of tenfold cross validation. Divide enrolled subjects rather than HS segments into tenfolds.

This procedure is illustrated in Figure 14, where 50 times of tenfold cross validation are conducted.

Thirdly, as noted in [156, 157], computational transportability is a critical issue when applying machine learning methods. By computational transportability of a computer-aided auscultation system, it means the possibility for other experts or clinicians in different institutions to apply the proposed system to their own dataset or patients. Studies on the automatic HS analysis addressing this issue are rare. The HS classifier is said to be highly transportable if it can be directly applied by users in another hospital or another lab without any further training process. Unfortunately, this might not be the case in every system or in every application. For example, if the classifier is to be applied to a different racial group, re-training might need to be conducted to cater for differences in physiology and anatomy in the different population groups, which limits its use as a universal diagnostic device. One possible solution is to build a model that allows the system to perform well when transferred to new institutions with minimal tuning through re-training. For example in [158], a layered HMM representation has been formulated with the ability to decouple different levels of analysis for training and inference. Each level of the hierarchy is trained independently, with different feature vectors and time granularities. In consequence, the lowest, signal-analysis layer, that is most sensitive to new conditions, can be retrained to tune the parameters, while leaving the higher-level layers unchanged. Another issue related to transportability is the reproducibility of the results. Unlike conventional statistical model, machine learning systems are usually less transparent and poorly documented. Moreover, the system might produce different results if implemented in different software environments, which further makes it less reproducible [157]. Hence, it is highly recommended that the software codes are properly documented and the implementations of the method are made publicly available.

Fourthly, fairly extensive and well-designed clinical trials have to be conducted, in order for the computer-aided auscultation system and device to get approval from FDA, and to be accepted by the medical professionals. Although preliminary results are promising, the clinical evaluation procedures of HS classification systems still have limitations. Most of the studies have been performed in the laboratory environment and the system has been tested with a limited set of data. In [105], a very preliminary study was conducted on the automatic HS analysis to classify AS, AR, MS and MR. Therein, 250 cardiac periods of HS data from HS simulator were used for system training and testing. Even though encouraging results were reported (with sensitivity and specificity of 97.5 and 100%), validations with larger sets of real HS data are definitely necessary to produce more reliable and stable performance evaluation. Furthermore, to determine the real clinical usefulness of the computer-aided auscultation system, it would be required to have a controlled clinical trial with blinded method. Table 6 gives the clinical trials with electronic stethoscope listed in the ClinicalTrials.gov registry. In [159], the cardiac sonospectrographic analyzer (CSA) output and the CT data of 200 patients were prospectively analyzed and compared in a double-blinded manner. The overall sensitivity of 89.5% and specificity of 57.7% for the CSA to predict coronary artery disease were statistically significant. Similarly in [160], the AS acceleration indices obtained from HS data for 50 patients were correlated double blindly to standard transthoracic echo measurements to predict the severity of AS.

**Table 6:** Clinical trials with electronic stethoscope listed in the ClinicalTrials.gov registry

References and ClinicalTrials.gov identifier	Title	Study population	Enrolment	Results
[159] NCT01040923	Validation of the cardiosond sonospectrographic digital electronic stethoscope in diagnosing coronary artery disease versus CT angiography	Patients presenting themselves for cardiac CT angiography	200	Sensitivity: 89.5%; Specificity: 57.7%;
[160] NCT01605669	Correlation of auscultatory severity of aortic stenosis with trans thoracic echocardiography	Patients with aortic stenosis seen in the internal medicine clinic	50	Sensitivity: 85.7%; Specificity: 72.4%; Accuracy: 75%
[161] NCT01665820	Study EM-05-012530 benefit of auscultation with 3 M™ Littmann 3200 electronic stethoscope to diagnose murmurs and heart pathologies in overweight and obese patients with increased layers of adipose tissue	Patients with a BMI greater than 30 and who have been scheduled for an echo examination	30	NM
[162] NCT00564122	A comparison of the accuracy of an artificially-intelligent stethoscope versus pediatric cardiologists in the assessment of pediatric patients referred to a cardiologist for the assessment of a heart murmur	Patients in outpatient pediatric cardiology clinic site	300	NM
[163] NCT01512927	Electronic stethoscope and computer-aided diagnostic system in detecting arteriovenous fistula stenosis	Patients with regular hemodialysis and who have received angioplasty	150 (expected)	Sensitivity: 86.2%; Specificity: 95.2%; Accuracy:>90%

*NM* not mentioned.

Lastly, in most of the present research work, the diagnosing process of cardiac dysfunction with HS is usually performed offline. The recorded HS data is stored locally and transferred to a PC for signal interpretation. Here two approaches could be considered to further improve the electronic stethoscope system:

- Stethoscope with embedded autonomous analysis and interpretation of HS. The signal processing including noise suppression and event recognition can be implemented by using a low power digital signal processor. With this embedded system design, the stethoscope can become not only valuable to the physician but also simple for home use by the patients themselves, who are usually not considered as the target user of the stethoscope.
- Stethoscope coupled with a hosting device or a server for sophisticated analysis (coupled to a PC with a Bluetooth link) for the use of professionals, in order to improve performance of clinical medical diagnosis.

The development and commercialization of real-time computer based HS analysis system will be a major area for future research approaches.

Comprehensive suggestions for future development of computer-aided auscultation system from the cardiologist’s point of view have been presented in [152]. Some key suggestions therein are summarized below:

- The system must be easy to use by minimally trained personnel in a primary healthcare setting.
- The automatic signal analysis must be performed in real-time to allow for immediate patient disposition.
- Simultaneous HS data recording at multiple chest sites with multiple sensors are more beneficial in terms of diagnostic accuracy, since the results from a single HS signal can be cross-referenced with those obtained from other sites.

## Conclusions

Cardiac auscultation constitutes an important part of clinical medicine. Recently, techniques associated with advances in electronic stethoscope have been developed to process auscultatory HS signals as well as analyze and clarify the resulting sounds, to make diagnosis based on quantifiable medical assessment. The availability of automatic interpretation of HSs opens interesting perspectives in the context of cardiovascular diagnostic measures.

The stethoscope is used by each and every doctor and nurse. So if we can incorporate into the electronic stethoscope (Figures 3, 12 and 13), the technology of automated detection of cardiac disorders (Figure 4), then it will become even more useful. As soon as the doctor applies the stethoscope, right away will s/he be able to know the disorders.

This comprehensive paper is designed to review recent technological advances, summarize promising innovations and perspectives in the field of computer-aided HS processing and analysis, investigate the gaps between technology and its real clinical application, and suggest probable areas for future research to mature the technology and to unlock the clinical value.

## Abbreviations

ALE: adaptive line enhancer
ANC: adaptive noise canceller
ANN: artificial neural network
AR: aortic regurgitation
AS: aortic stenosis
CMOS: complementary metal-oxide-semiconductor
CSA: cardiac sonospectrographic analyzer
CT: computed tomography
DWT: discrete wavelet transform
ECG: electrocardiogram
Echo: echocardiogram
EEMD: ensemble empirical mode decomposition
ELM: extreme learning machine
EMD: empirical mode decomposition
ES: ejection sound
FDA: food and drug administration
FFT: fast Fourier transform
FT: Fourier transform
GMM: Gaussian mixture model
HF: High frequency
HMM: Hidden Markov model
HOCM: hypertrophic cardiomyopathy
HS: Heart sound
ICA: Independent component analysis
ICS: intercostal space
IIR: infinite impulse response
IMF: intrinsic mode function
KNN: k-nearest neighbors
LF: low frequency
LMS: least mean square
LS: lung sound
MEMS: micro-Electro-Mechanical System
MFCC: mel frequency cepstral coefficients
mHealth: mobile health
MMSE: Minimum mean square error
MP: Matching pursuit
MR: mitral regurgitation
MRI: magnetic resonance imaging
MS: mitral stenosis
MSC: mid-systolic click
MVP: mitral valve prolapse
NHS: normal heart sound
NLMS: normalized least mean square
PCG: phonocardiogram
PDA: patent ductus arteriosus
PR: pulmonary regurgitation
PS: pulmonary stenosis
PSD: power spectral density
RLS: recursive least square
S1: the first heart sound
S2: the second heart sound
S3: the third heart sound
S4: the fourth heart sound
SG: summation gallop
SIANC: single input adaptive noise canceller
S-LMS: subband least mean square
S-NLMS: Subband normalized least mean square
SNR: signal-to-noise ratio
SS: spectral subtraction
STFT: short time Fourier transform
STSA: short-time spectral amplitude
SURE: Stein’s unbiased risk estimate
SVM: support vector machine
TR: tricuspid regurgitation
TS: tricuspid stenosis
VHD: valvular heart disease
VSD: ventricular septal defect
WT: wavelet transform
