# Correction: The electronic stethoscope

**DOI:** 10.1186/s12938-025-01457-7

**Published:** 2025-10-09

**Authors:** Shuang Leng, Ru San Tan, Kevin Tshun Chuan Chai, Chao Wang, Dhanjoo Ghista, Liang Zhong

**Affiliations:** 1 National Heart Research Institute Singapore, National Heart Centre Singapore, 5 Hospital Drive, Singapore, 169609 Singapore; 2 Cardiovascular and Metabolic Disorders Program, Duke-NUS Graduate Medical School, 8 College Road, Singapore, 169857 Singapore; 3 Institute of Microelectronics, A*STAR, 11 Science Park Road, Singapore, 117685 Singapore; 4 University 2020 Foundation, Northborough, MA USA

**Correction: BioMed Eng OnLine (2015) 14:66** 10.1186/s12938-015-0056-y

In Table 1 of this article [1], entries for Tricuspid regurgitation (TR) in the Location and Timing columns were incorrect. The both incorrect and correct entries are given below.

Incorrect table entry

**Table:** 

Murmur type	Location	Timing	Pitch	Quality
Tricuspid regurgitation (TR)	Left fourth ICS	Diastolic	High	Blowing

Correct table entry

**Table:** 

Murmur type	Location	Timing	Pitch	Quality
Tricuspid regurgitation (TR)	Left lower sternal border	Systolic	High	Blowing
